# Heterogeneous Formation of Organonitrates (ON) and Nitroxy-Organosulfates (NOS) from Adsorbed α-Pinene-Derived Organosulfates (OS) on Mineral Surfaces

**DOI:** 10.1021/acsearthspacechem.2c00259

**Published:** 2022-11-29

**Authors:** Eshani Hettiarachchi, Vicki H. Grassian

**Affiliations:** Department of Chemistry and Biochemistry, University of California San Diego, 9500 Gilman Drive, La Jolla, California 92093, United States

**Keywords:** α-pinene, SOA, nitroxy-organosulfates (NOS), organonitrates (ON), organosulfates (OS), iron oxide, kaolinite

## Abstract

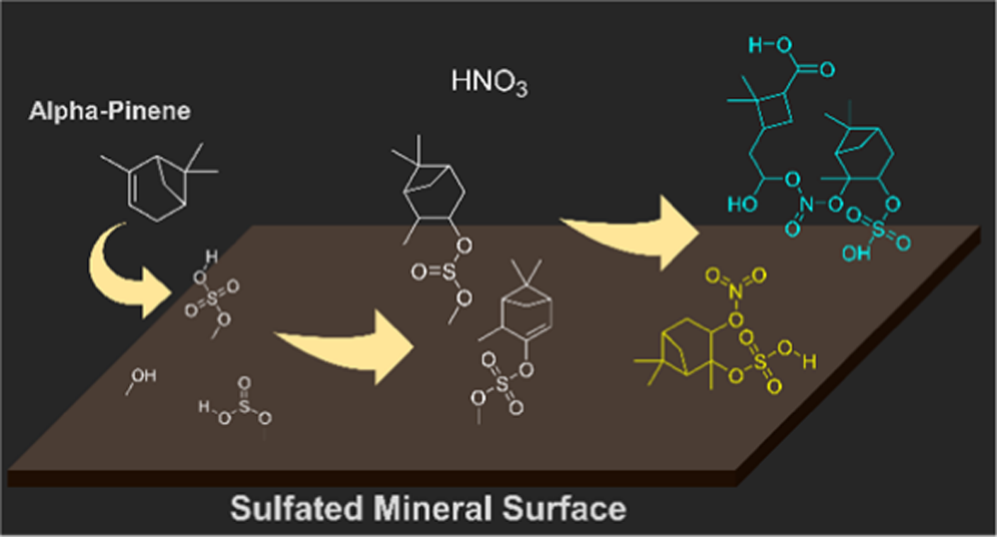

Organonitrates (ON) and nitroxy-organosulfates (NOS) are important components of secondary organic aerosols (SOAs). Gas-phase reactions of α-pinene (C_10_H_16_), a primary precursor for several ON compounds, are fairly well understood although formation pathways for NOS largely remain unknown. NOS formation may occur via reactions of ON and organic peroxides with sulfates as well as through radical-initiated photochemical processes. Despite the fact that organosulfates (OS) represent a significant portion of the organic aerosol mass, ON and NOS formation from OS is less understood, especially through nighttime heterogeneous and multiphase chemistry pathways. In the current study, surface reactions of adsorbed α-pinene-derived OS with nitrogen oxides on hematite and kaolinite surfaces, common components of mineral dust, have been investigated. α-Pinene reacts with sulfated mineral surfaces, forming a range of OS compounds on the surface. These OS compounds when adsorbed on mineral surfaces can further react with HNO_3_ and NO_2_, producing several ON and NOS compounds as well as several oxidation products. Overall, this study reveals the complexity of reactions of prevalent organic compounds leading to the formation of OS, ON, and NOS via heterogeneous and multiphase reaction pathways on mineral surfaces. It is also shown that this chemistry is mineralogy-specific.

## Introduction

Secondary organic aerosols (SOA) are ubiquitous in the atmosphere and are known to play key roles in climate change, air quality, reduced visibility, and human health.^1−5^ Organosulfates (OS, ROSO_3_H), organonitrates (ON, RONO_2_), and nitroxy-organosulfates (NOS, RN*_x_*S*_y_*O*_n_*) are present within SOA and represent a structurally diverse mixture of compounds found in polluted environments.^6−10^ Precursors of these compounds include both biogenic and anthropogenic sources of organic compounds. Primary biogenic precursors are isoprenes, monoterpenes, sesquiterpenes, and aldehydes.^11^

α-Pinene (C_10_H_16_), a biogenic volatile organic compound, is one of the most abundant atmospheric monoterpenes with an average estimated emission of 66 Tg yr^–1^.^12−15^ Furthermore, α-pinene-derived OS-, ON-, and NOS-SOA along with oxygenated α-pinene derivatives have been detected in ambient air.^16−19^ The formation of OS compounds in the atmosphere occurs via several reaction pathways. OS is produced from the photochemical reaction of biogenic hydrocarbons under highly acidic conditions, such as with sulfate aerosols or sulfuric acid, via nucleophilic substitution reactions of sulfate with epoxides in aqueous-phase solutions, with HO radical-involved mechanisms, and via catalytic reactions with photocatalysts such as TiO_2_.^16,17,20−25^ Schmidt et al. showed increased formation of OS from methacrolein under illuminated conditions in the presence of road dust.^25^ Numerous laboratory studies have been conducted to understand the formation of OS from α-pinene.^22,24,26,27^ Surratt et al. reported that the OS is formed when monoterpenes are oxidized in the presence of acidified sulfate aerosol.^22^

Despite the fact that atmospheric OS represents a significant portion of the organic aerosol mass, estimated to be between 3 and 30%,^18,28,29^ the formation of ON and NOS from reactions of OS due to nighttime heterogeneous and multiphase chemistry is less understood. Atmospheric ON formation occurs either by OH radical-initiated photochemical reactions or NO_3_ radical-initiated nocturnal reactions of anthropogenic and biogenic volatile organic compounds.^30−35^ Reactions of α-pinene with strong atmospheric oxidizers such as O_3_ and OH radicals lead to the formation of a range of oxidized α-pinene derivatives that are known as first-generation oxidation products.^4,12,36,37^ Some of these compounds are pinonaldehyde, pinonic acid, α-pinene oxide, and structurally diverse organic peroxides. These oxidation products can also act as a reactant in generating atmospheric OS and ON compounds.^38^ Oxidation reactions generally occur through photochemical pathways in the presence of sunlight.^30,39^ Recently, it was shown that the formation of ON from α-pinene via heterogeneous reactions on mineral surfaces under dry and dark conditions can occur.^40^ In the case of NOS formation, its presence in aerosols is almost always observed in the night time. Surratt et al.^22^ suggested NOS formation from the reactions of ON with sulfates under acidification, whereas Iinuma et al.^17^ suggested the formation of some common NOS such as C_10_H_17_NSO_7_ and C_10_H_18_NSO_7_ occurs during night time. Similar to OS, NOS formation may occur through reactive uptake of epoxides, esterification of hydroxyl and keto groups with sulfuric acid,^41^ and/or through radical-initiated processes in wet aerosols.^42^ Increased NOS formation due to fireworks has also been previously observed.^11^ However, formation pathways for NOS, in general, are less well understood, although their relative abundance and formation pathways involving heterogeneous chemistry in nighttime conditions remain scarce.

In this study, reactions of NO_2_ and HNO_3_ with α-pinene-OS adsorbed on mineral surfaces hematite (α-Fe_2_O_3_) and kaolinite (SiO_2_Al_2_O_3_(OH)_4_) were studied. The adsorbed α-pinene-OS was produced by exposing α-pinene on mineral surfaces, hematite and kaolinite, first exposed to SO_2_. Hematite and kaolinite are major reactive components of mineral dust.^43^ Mineral surfaces can interact with gas-phase SO_2_,^5,44,45^^5,44,45^ leading to the formation of surface products.^46,47^ In fact, the interaction of SO_2_ on mineral surfaces has been widely studied^48−50^ and it has been shown that several surface-adsorbed species form, including sulfate, bisulfate, sulfite, and bisulfite, thereby producing a surface for α-pinene to interact with and facilitating the formation of OS. Additionally, both NO_2_ and HNO_3_ are found in trace quantities in the atmosphere, primarily via fossil fuel combustion, vehicle exhausts, and agricultural activities.^45−47,51^ Due to the higher correlation of atmospheric NO_2_ concentrations to traffic, it is used as a traffic-related air pollution marker.^46^ Therefore, interactions between α-pinene with mineral dust and trace gas pollutants during dust transport and urban dust episodes can facilitate formation of OS, ON, and NOS.

Given the importance of heterogeneous reactions of atmospheric gases with mineral dust surfaces in the atmosphere and due to the abundance of α-pinene, the role of mineral dust surface chemistry in reactions of α-pinene-OS with atmospheric NO_2_ and HNO_3_ on hematite and kaolinite surfaces was investigated. This study represents a step forward in understanding the complexity of reactions of volatile and semivolatile organics with trace inorganic gases on mineral surfaces to form multifunctional compounds that can modify the climate impacts of mineral dust.^52^ In the current study, experiments were conducted under dark and dry conditions. Although water vapor and adsorbed water play an important role in the chemistry of these surface-mediated reactions,^53^ these initial studies were conducted in the absence of coadsorbed water. Both Fourier transform infrared (FTIR) spectroscopy as an in situ probe of product formation and high-resolution mass spectrometry (HRMS) of solvent-extracted products were used to unravel the complex heterogeneous chemistry occurring on mineral dust surfaces.

## Materials and Methods

### Transmission FTIR Experiments and Methods

Transmission FTIR spectroscopy was used to monitor reactions on mineral surfaces at 296 ± 1 K. Additional details of the infrared cell and gas handling system have been previously described.^40,54−58^ Mineral particles hematite (α-Fe_2_O_3_, 99+%, Fischer Scientific) or kaolinite (SiO_2_Al_2_O_3_(OH)_4_, Sigma-Aldrich) with a BET surface area of 80 ± 10 and 8.4 ± 0.5 m^2^/g, respectively, were heated in an oven at 473 ± 1 K overnight to remove organic contaminants and then pressed onto one half of a tungsten grid (ca. 5 mg). The grid was then placed in the sample IR cell compartment held by two stainless steel jaws. Following the preparation of the mineral sample and placement in the IR cell, the system was evacuated for 4 h using a turbomolecular pump. Mineral surfaces were subsequently exposed to 50% RH water vapor for 2 h to ensure a fully hydroxylated terminated surface. Once hydroxylated, the system was evacuated for another 6 h to remove water vapor in the chamber. After evacuation, the sample was exposed to 100 mTorr of SO_2_ (99+%, Sigma-Aldrich) for 20 min under dry conditions (RH < 1%) to obtain an SO_2_-exposed mineral surface. The chamber was then evacuated for 4 h to remove gas-phase SO_2_ and any weakly bonded SO_2_ on the surface. This evacuation ensures that the incoming α-pinene interacts with only strongly adsorbed SO_2_ species, and not with any weakly adsorbed SO_2_ species. After evacuation, the sample was exposed to 500 mTorr of α-pinene (99+%, Sigma-Aldrich) for 20 min under dry conditions (RH < 1%), and the formation of OS on the surface was studied. The α-pinene sample was degassed at least three times with consecutive freeze–pump–thaw cycles prior to use.

Reactions of gas-phase HNO_3_ from the nitric acid vapor taken from a concentrated mixture of H_2_SO_4_ (∼96 wt %)–HNO_3_(∼70 wt %) 3:1 ratio^59^ and NO_2_ (26.5 ppm in N_2_, Airgas) with adsorbed α-pinene-OS on mineral surfaces were studied. First, OS was produced on the surface as described above. The desired pressure of HNO_3_ (50 mTorr) or NO_2_ (7 mTorr) was then introduced into the IR cell. FTIR spectra were collected over a 4 h period through both halves of the tungsten grid to monitor the gas phase and particle phase. Following adsorption, the system was evacuated overnight. The experiments with HNO_3_ were conducted in a different experimental but very similar setup, and instead of stainless steel a Teflon-coated cell was used. Relatively higher concentrations of reactants compared to their atmospheric concentrations were used in this study to determine the feasibility of these types of reactions. Additionally, mass spectroscopic analysis of surface products required these higher concentrations for definitive confirmation as lower concentrations yielded similar HRMS patterns with much lower intensity and higher mass error, suggesting the use of higher concentrations may not impact product formation beyond forming more products.

Prior to and following the exposure to gases, single-beam spectra (250 scans) of the surface and gas phase were acquired using a resolution of 4 cm^–1^ and covering the spectral range of 600–4000 cm^–1^. This was accomplished by the use of a linear translator with the infrared beam interrogating the portion of the grid pressed with mineral particles followed by the portion of the grid left blank. Absorption spectra on mineral particles are reported as the difference in the mineral spectra before and after exposure to gases. Absorption bands due to gas-phase components, as measured through the blank half of the tungsten grid under identical conditions, were subtracted to obtain FTIR spectra of adsorbed species only.

### HRMS Experiments and Methods

Organic products formed on mineral surfaces following reactions of α-pinene with inorganic gases (SO_2_, NO_2_, and HNO_3_) were analyzed using a direct-injection linear ion trap (ThermoFisher Orbitrap) high-resolution mass spectrometer (HRMS). Adsorbed products were extracted from the hematite or kaolinite solid substrate using methanol (CH_3_OH, Fisher Scientific, HPLC grade) as the solvent. Methanol was chosen as the solvent as it produced the best signal for α-pinene standards and OS products relative to other solvents and solvent mixtures (e.g., acetonitrile, ethanol, and their 1:1 mixtures with H_2_O). The sample vial, syringe, and all other glassware used in the transfer process were cleaned prior to use with methanol, and Milli-Q water (Millipore Sigma, 18.2 MΩ), and baked in an oven at 773 ± 1 K to further remove trace organics. Plastic vials used in sample preparation were sonicated in methanol for 60 min and washed thoroughly prior to use. All of the samples were stored at 253 ± 1 K and analyzed within 48 h of collection. Product stability was tested for up to 10 days in the freeze storage conditions used. It was determined that no transformations of products occurred. Extraction in different solvents confirmed no additional products were formed during the extraction step.

HRMS analysis in both positive electrospray ionization (ESI) ([M + H]^+^ and [M + Na]^+^) and negative ESI modes ([M – H]) was used. The heated electrospray ionization (HESI) source was operated at 373.15 K. The ESI capillary was set to a voltage of 3.5 kV at 623.15 K. The HESI-Orbitrap MS was calibrated prior to use. Mass spectra were acquired with a mass range of 50–2000 Da. Peaks with a mass tolerance of >5 ppm were rejected. Compositions were calculated with the following element ranges: 12C, 0–60; 1H, 0–150; 16O, 0–25; 14N, 0–5; 32S, 0–5; 23Na, 0–5; 39K, 0–5; 56Fe, 0–5. Tandem mass spectrometry (MS/MS) with a collision energy of 40 eV was used for structure determination.

## Results and Discussion

### Formation of Organosulfates from α-Pinene on Mineral Surfaces

These experiments were initiated by preparing mineral surfaces with adsorbed OS compounds. This was done by first exposing mineral surfaces to gas-phase SO_2_ followed by the exposure of α-pinene as described in the Materials and Methods section. For an SO_2_-exposed hematite surface, several species and coordination modes of adsorbed SO_2_ species have been observed (Figure S1 and Table S1). In the presence of gas-phase SO_2_, these species include molecularly adsorbed SO_2_, monodentate and bidentate adsorbed sulfite, adsorbed HSO_3_ and SO_2_·H_2_O complexes, and bidentate and bridging sulfate were observed.^48,60−62^ Following evacuation of gas-phase SO_2_, previous studies have shown that the adsorption of SO_2_ on hematite and other metal oxides forms both adsorbed sulfite and sulfate that remain strongly bound to the surface.^50,62^ Similarly, for a sulfated kaolinite surface, adsorbed sulfate species were observed.

**Figure 1:**
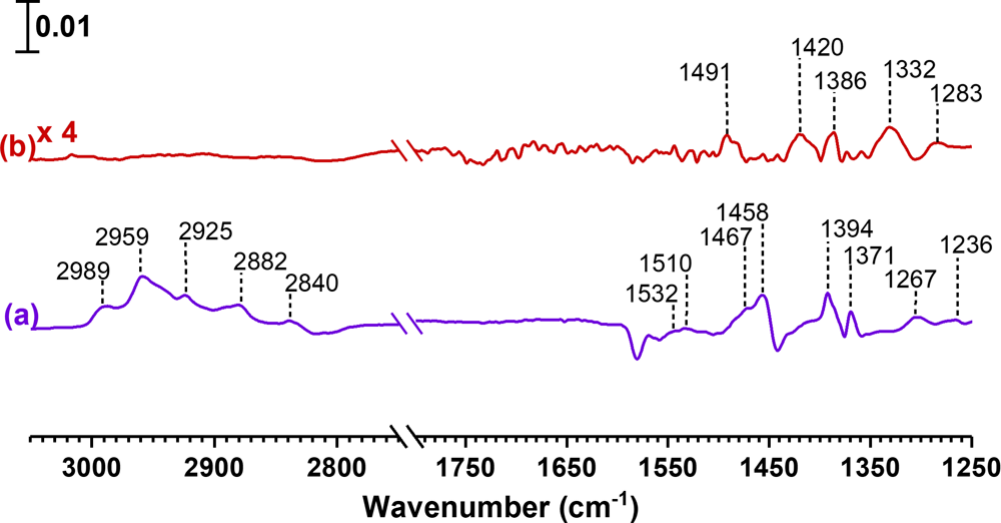
FTIR spectra of surface-bound products from the reaction of α-pinene on (a) sulfated hematite and (b) sulfated kaolinite in the spectral regions from 1250 to 1800 and from 2750 to 3050 cm^–1^. The absorbance scale is shown in the top left corner.

Upon exposure to α-pinene, the spectral features for α-pinene on these surfaces become apparent. After evacuation, surface-adsorbed products were retained on the surface (Figure 1). As shown previously, α-pinene weakly adsorbs on hematite and undergoes reversible adsorption. Thus, adsorption of α-pinene alone on hematite does not yield any strongly adsorbed product formation. In the case of kaolinite surfaces, it has been shown that α-pinene undergoes oxidation reactions on this surface to yield pinonaldehyde and pinonaldehyde dimer.^40^ For hematite, following exposure of SO_2_ and α-pinene, there remain adsorbed products following evacuation as evident by the peaks seen in the infrared spectrum. Several features were prominent in the following spectral range: from 2800–3000 cm^–1^ (C–H stretch); 1300–1500 cm^–1^ (C–H bond bending modes); and ∼1236 and 1267 cm^–1^ (C–O bond stretch). A concomitant loss of surface hydroxyl groups was observed between 3500 and 3700 cm^–1^ similar to when SO_2_ alone was adsorbed. For kaolinite, there were also peaks observed in the spectra albeit of weaker intensity. The most evident ones were from 1250 to 1550 and 3600 to 3900 cm^–1^. However, given the complexity of products formed on the surface, it is difficult to assign these FTIR spectra to specific molecules. Generally, covalent organosulfates have two strong bands, one at 1415–1370 cm^–1^ and the other at 1200–1185 cm^–1^, both of which are due to the stretching vibrations of the SO_2_ group.^63^ The peaks arising due to adsorbed sulfate and sulfite overlap in the same region as OS bond vibrations.^64^

**Figure 2:**
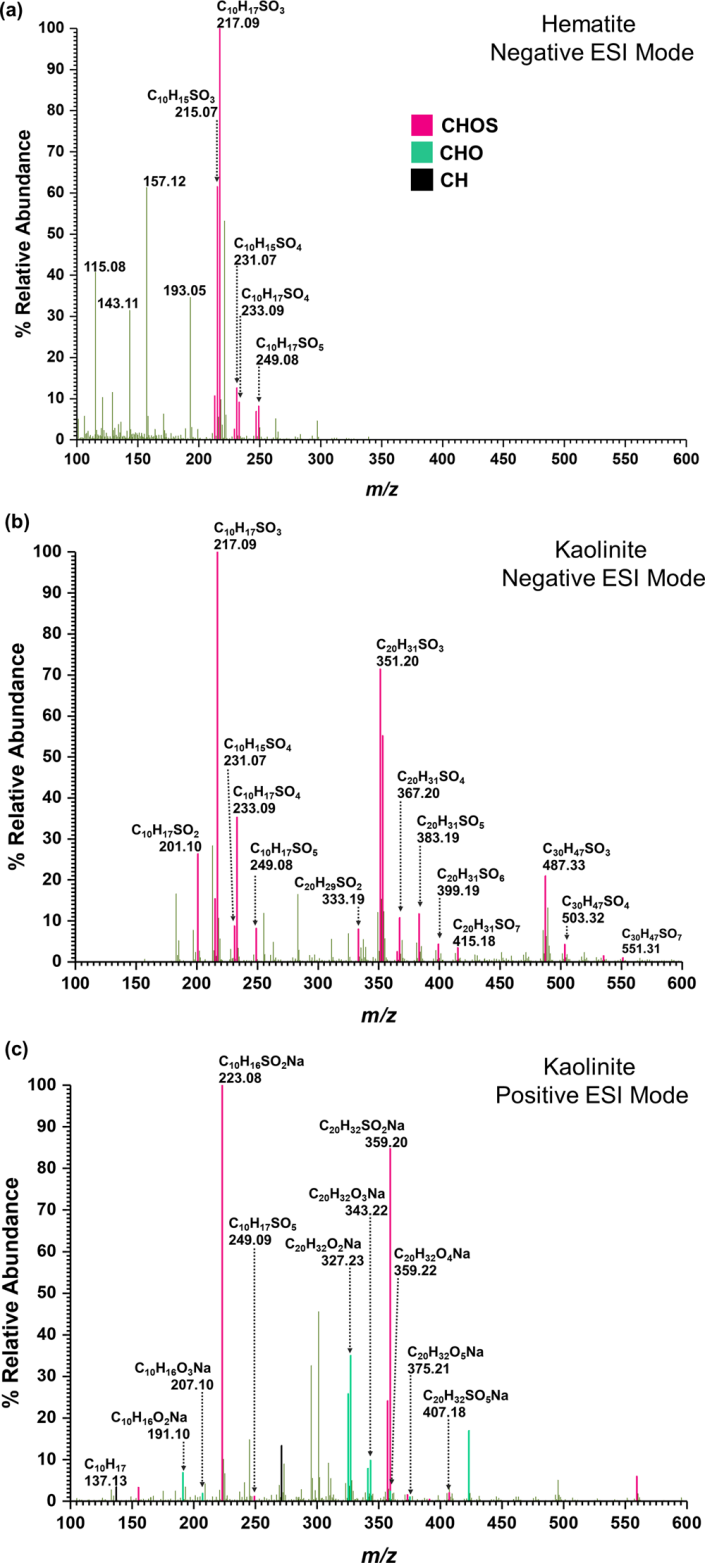
HRMS patterns in both positive [M + H]^+^ and negative [M – H]^−^ ESI modes, of surface-bound products from the reaction of α-pinene with sulfated mineral surfaces on (a) hematite and (b, c) kaolinite. HRMS pattern for hematite in positive-ESI mode did not produce any analyte peaks. Confirmed peaks are color-coded according to heteroatoms. Pink box solid—CHOS; green box solid—CHO; black box solid—CH.

To obtain additional molecular-level insights into these surface-bound products, compounds were solvent-extracted from the surface and further analyzed for product identification using HRMS (Figure 2). The HRMS patterns of surface products collected in neg-ESI mode from the reactions of α-pinene with adsorbed SO_2_ species on hematite indicated the presence of several OS compounds (Figure 2 and Table 1).

**Table 1:**
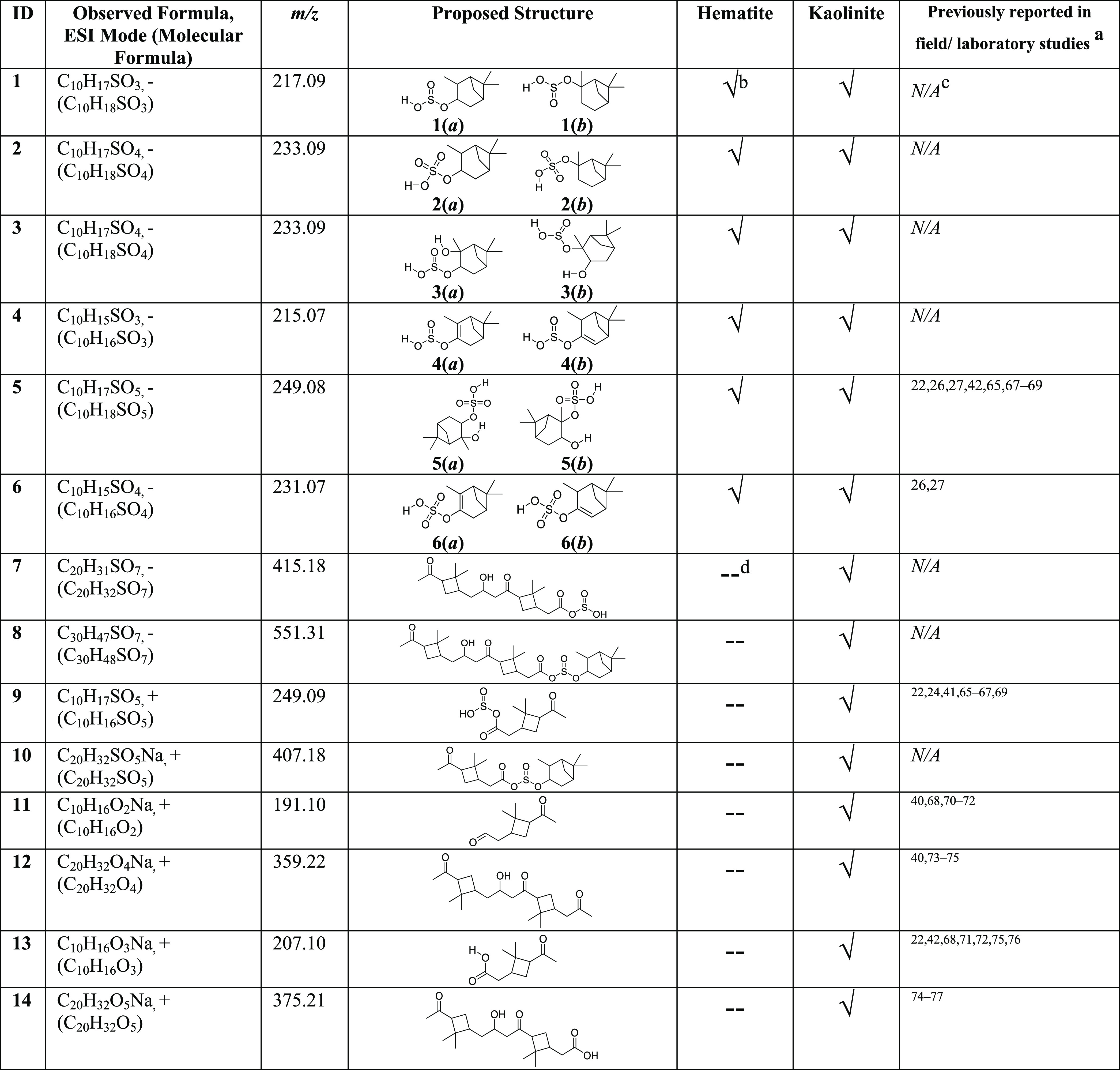
Identified Surface Products Using HRMS and MS/MS in Either Positive [M + H]^+^ or Negative ESI [M – H]^−^ Modes from the Reactions of α-Pinene on Sulfated Mineral Surfaces^a^^b^^c^^d^

a One or more of the compound, molecular formula, and *m/z* has been reported.

b √ = Detected in HRMS.

c *N/A* = Not available.

d -- = Not detected in HRMS.

These identified OS compounds correspond to C_10_H_17_SO_3_ (compound **1**, *m*/*z* = 217.09), C_10_H_17_SO_4_ (compounds **2** and **3**, *m*/*z* = 233.09), C_10_H_15_SO_3_ (compound **4**, *m*/*z* = 215.07), C_10_H_17_SO_5_ (compound **5**, *m*/*z* = 249.08), and C_10_H_15_SO_4_ (compound **6**, *m*/*z* = 231.07). The structures of these compounds were confirmed via tandem mass spectrometry. Furthermore, neither oxygenated α-pinene derivatives nor unreacted α-pinene were observed via HRMS analysis. For kaolinite, several OS compounds were identified from both neg- and pos-ESI modes. In neg-ESI mode, apart from compounds **1**–**6**, two new OS were identified. These are C_20_H_31_SO_7_ (compound **7**, *m*/*z* = 415.18) and C_30_H_47_SO_7_ (compound **8**, *m*/*z* = 551.31). In pos-ESI mode, C_10_H_17_SO_5_ (compound **9**, *m*/*z* = 249.09) and C_20_H_32_SO_5_Na (compound **10**, *m*/*z* = 407.18) along with pinonaldehyde (C_10_H_16_O_2_Na, compound **11**, *m*/*z* = 191.10), pinonalehyde dimer (C_20_H_32_O_4_Na, compound **12**, *m*/*z* = 359.22), pinonic acid (C_10_H_16_O_3_Na, compound **13**, *m*/*z* = 207.10), and C_20_H_32_O_5_Na (compound **14**, *m*/*z* = 375.21) were observed. The presence of both compounds **2** and **3** was confirmed via MS/MS analysis. Similarly, the structure for **9** was determined based on its major fragments at *m*/*z* = 223.08 (C_10_H_16_SO_2_Na) and 191.10 (C_10_H_16_O_2_Na), which suggested the presence of a pinonaldehyde moiety. Table S2 provides detailed information on the mass fragmentation of identified compounds. Similar OS compounds were identified in various field and chamber studies, suggesting the formation of these OS compounds from α-pinene occurs in a broader environment.^19,26,27,41,65−67^

**Scheme 1:**
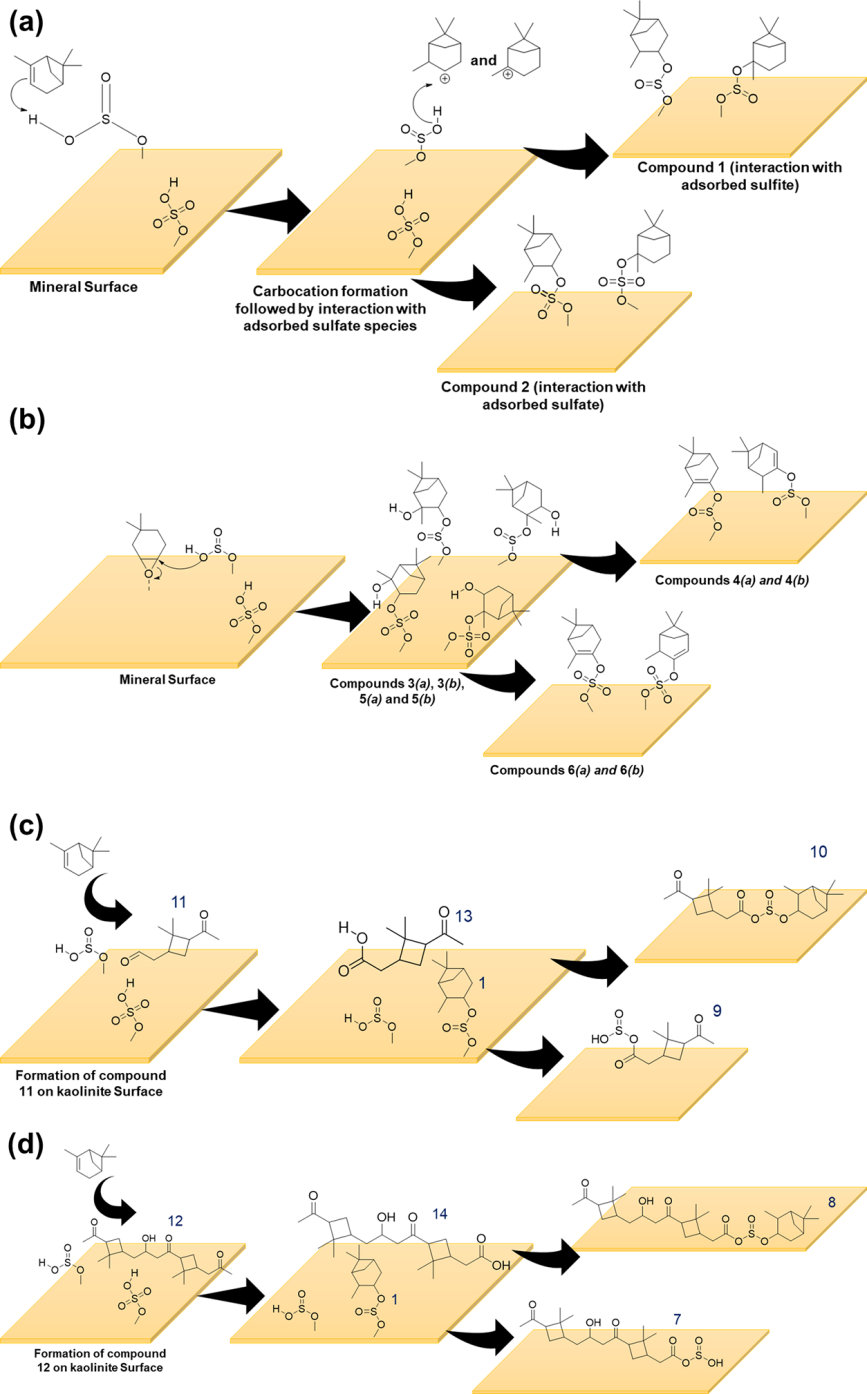
Proposed Formation Pathways for Several of the Compounds Observed in HRMS Compounds (a) **1** and **2**, (b) **3**, **4**, **5**, and **6** on Mineral Surfaces and (c) **9**, **10**, **11**, and **13**, (d) **7**, **8**, **12**, and **14** on Kaolinite Surfaces

Formation pathways of these OS compounds are facilitated on these mineral surface mechanisms (Scheme 1).^70,73−77^ Briefly, the adsorbed SO_2_ species on mineral surfaces can interact with π bonds of α-pinene in the gas phase, producing a series of OS compounds.^26,27,78,79^ Compounds **1** and **2** can form via carbocation formation from the reaction of α-pinene with adsorbed sulfate/sulfite on mineral surfaces. The reaction of α-pinene π bonds with acid groups such as sulfate was previously proposed.^17^ The carbocation can react with adsorbed sulfite or sulfate species on mineral surfaces to form the isomers of compounds **1** or **2**, respectively (Scheme 1a). Compounds **3** and **5** can form via the interaction of π bonds in α-pinene with the surface redox sites/surface hydroxyl groups on mineral surfaces, leading to the formation of pinene oxide-like species. A similar reaction is prominent in limonene (an isomer of α-pinene) forming limonene oxide;^80^^80^ here, the interactions are expected to be weaker for α-pinene given no α-pinene oxide was observed from its interactions with mineral surfaces.

The reaction of adsorbed sulfite or sulfate species on mineral surfaces with one of the two epoxide carbons on α-pinene can yield compound **3** or **5**. The presence of peaks corresponding to HSO_3_^–^ (*m*/*z* = 80.97), SO_3_ (*m*/*z* = 79.95), and HSO_4_^–^ (*m*/*z* = 96.96) in the HRMS fragmentation pattern of *m*/*z* = 233.09 further confirms the formation of both sulfate and sulfite groups containing α-pinene-derived OS compounds.

Two formation pathways are proposed for compound **4**. First, the dehydration of compound **3** yields compound **4**. Second, the loss of 2H atoms from compound **1** in the mass spectrometer can yield *m*/*z* = 215.07. Similarly, compound **6** can form from the dehydration of compound **5** or the removal of 2H atoms from compound **2** during the HRMS analysis (Scheme 1b). Dehydration of compounds **3** and **5** from compounds **4** and **6**, respectively, can occur either on mineral surfaces or during the HRMS analysis. Duporté et al. observed an OS at 249 (compound **5** in this study) dehydrating to an OS at 231 (compound **6**) during MS analysis.^27^

On kaolinite, α-pinene derivatives pinonaldehyde (**11**) and pinonaldehyde dimer (**12**) are formed^40^ and further oxidized to form their respective acids (compounds **13** and **14**). Formation of pinonaldehyde and pinonaldehyde dimer upon exposure to kaolinite surfaces is explained in detail in our previous study.^40^ Briefly, these are from the dihydroxylation of double bond on α-pinene followed by glycol cleavage on the kaolinite surface. Compound **7** can be expected to form from the reaction of adsorbed sulfites with compounds **11** and **12**, respectively. In this case, adsorbed sulfites act as a nucleophile and attack aldehyde carbon on either compound **9** or **7**. Compound **9** can also form from the olefinic cleavage of compound **4** in a similar way to pinonaldehyde formation from α-pinene. Compound **8** can be expected to form due to the reaction between compounds **14** and **1**. Similarly, compound **10** may be formed due to the reaction between compounds **13** and **1** (Scheme 1c,d). Therefore, these results show the formation of these multiple OS surface products from reactions of α-pinene with adsorbed SO_2_ species on mineral surfaces in dark and dry conditions, in the absence of strong atmospheric oxidizers such as HO radicals and O_3_, thus underscoring the unique role the surface plays. These compounds interact with adsorbed SO_2_ species on surfaces, yielding a more complex mixture of SOAs. One such possible interaction is the relatively well studied sulfate ester and OS formation from pinonaldehyde.^16,22,26,27,41,66^

### Reactions of Gas-Phase Nitric Acid and Nitrogen Dioxide with Adsorbed α-Pinene-OS

**Figure 3:**
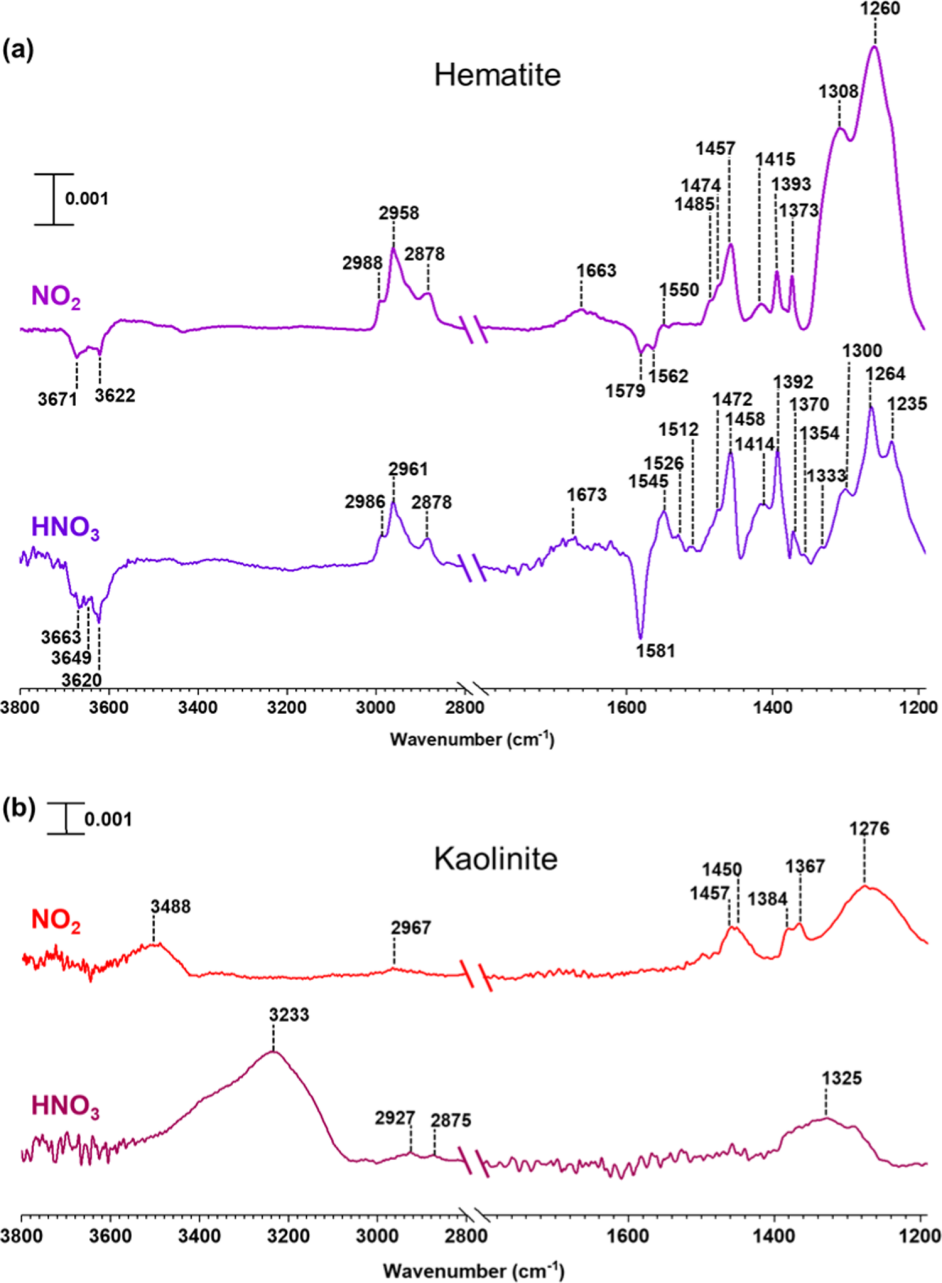
FTIR spectra of surface-bound products from the reaction of adsorbed α-pinene-OS with either HNO_3_(vapor) or NO_2_(g) on (a) hematite and (b) kaolinite in the spectral regions from 2800 to 3800 cm^–1^ and from 1190 to 1800 cm^–1^. The absorbance scale is shown in the upper left for each surface.

In these FTIR experiments, the mineral surfaces were prepared as described above to form adsorbed OS. These surfaces were then exposed to either gas-phase HNO_3_ or NO_2_ for several hours (4 h). The evacuated surfaces are shown in Figure 3. Upon exposure to HNO_3_, new spectral features around 1500–1550 and ∼1300 cm^–1^ started to appear on the hematite surfaces. These are attributed to different types of adsorbed nitrate species.^58^ Moreover, asymmetric and symmetric stretching vibrations of the NO_2_ group of ON occur at 1615–1660 and 1270–1285 cm^–1^.^63^ In addition to these, peaks at 2878, 2961, and 2986 cm^–1^ were attributed to C–H stretching modes, and peaks at 1472 and 1458 cm^–1^ were assigned to C–H bending modes. In contrast, for kaolinite surfaces, only weaker spectral features corresponding to organics at 2875, 2927, and ∼1325 cm^–1^ were observed. However, a strong band at 3233 cm^–1^ was observed, suggesting the presence of hydrogen-bonded networks that could include the presence of alcohols, and/or protonated carboxylic acids. Upon exposure to NO_2_, minimal changes of spectral features from OS adsorbed on hematite were observed at ∼1370–1420 cm^–1^, but a weak band at 1550 cm^–1^ was seen.

For kaolinite, spectral features around 1300–1450 cm^–1^ as well as weaker features at ∼2967 cm^–1^ were observed, suggesting the presence of C–H bonds on the surface. Furthermore, a strong band at 3488 cm^–1^ was observed, indicating the presence of O–H groups. However, given the complexity of products formed on the surface for specific compounds, these surface-bound products were extracted and analyzed using HRMS and MS/MS to identify products and for structural determination.

### Product Formation from the Reactions of Adsorbed α-Pinene-OS with Gas-Phase HNO_3_

**Figure 4:**
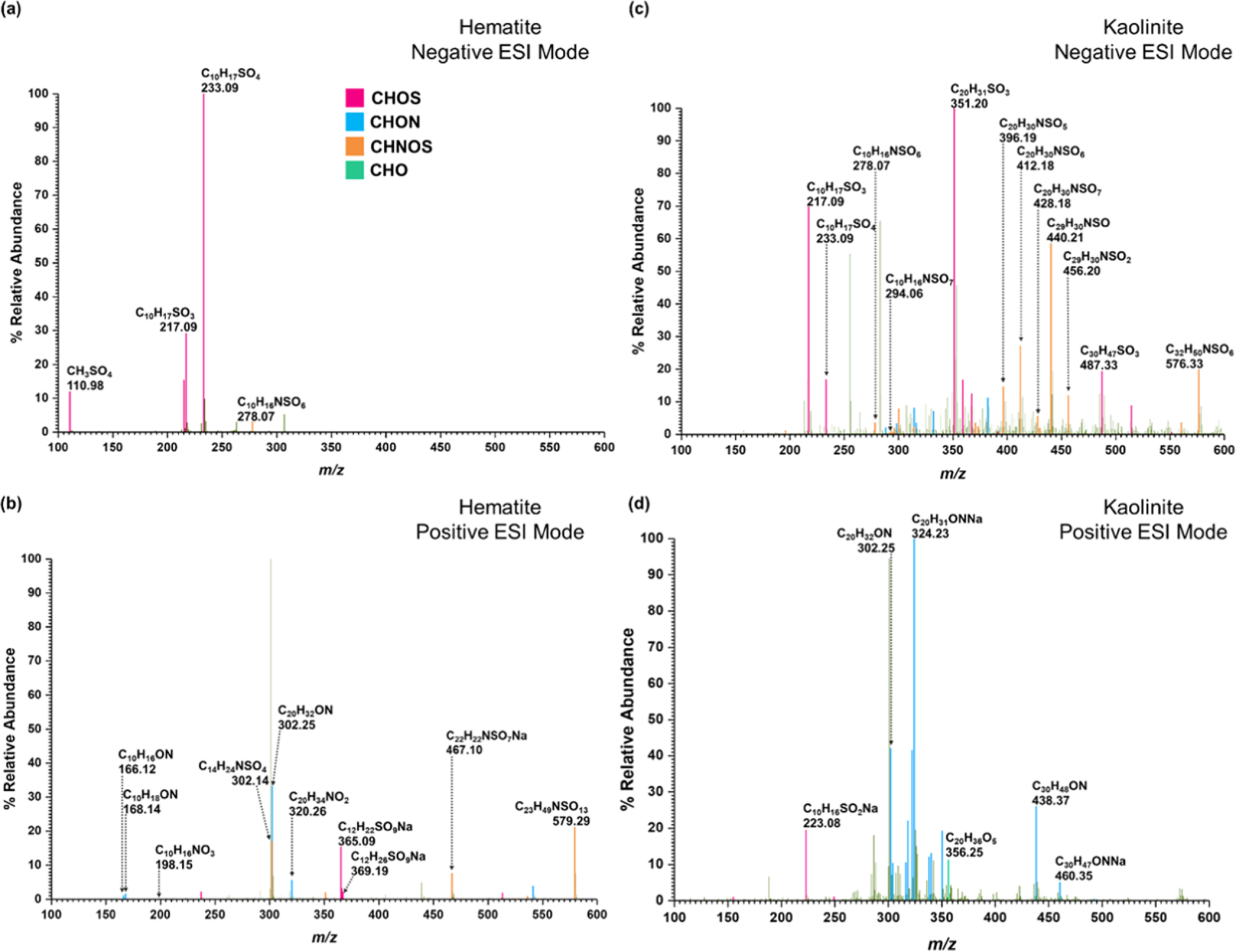
HRMS patterns in both positive [M + H]^+^ and negative [M – H]^−^ ESI modes of surface-bound products from the reactions of adsorbed α-pinene-OS with HNO_3_(vapor) on (a, b) hematite and (c, d) kaolinite. Confirmed peaks are color-coded according to heteroatoms. Pink box solid—CHOS; blue box solid—CHON; orange box solid—CHNOS; green box solid—CHO; black box solid—CH.

The HRMS patterns collected in both neg- and pos-ESI modes of surface products from reactions of HNO_3_ with adsorbed OS species on hematite indicated the presence of several OS compounds, ON compounds, and NOS compounds (Figure 4a,b, Table 2). Apart from the presence of compounds **1** and **2**, a new OS, Compound **15** (C_9_H_14_SO_6_, sulfated pinalic-3-acid, *m*/*z* = 369.12 for C_12_H_26_SO_9_Na) was observed. Compound **15** was observed as a sodated cluster of three methanol units and with a major fragment at 365.09 for C_12_H_22_SO_9_Na. Furthermore, *m*/*z* = 233.09 was assigned only to compound **2**, as no indication of SO_3_ was shown on its MS/MS fragmentation. Two major ON products were identified. Compound **16** (C_10_H_16_NO_3_, *m*/*z* = 198.15) was identified with its major fragment 166.12 for C_10_H_16_ON. Compound **17** (C_20_H_34_NO_3_, *m*/*z* = 336.25) was identified with major fragments at 320.26 for C_20_H_34_NO_2_, 302.25 for C_20_H_32_ON, and 168.14 for C_10_H_18_ON. Two NOS were identified. Compound **18** (C_10_H_16_NSO_7_, *m*/*z* = 294.06) was identified with a major fragment at 278.07 for C_10_H_16_NSO_6_. Compound **19** (C_19_H_32_NSO_10_, *m*/*z* = 595.29 for C_23_H_49_NSO_14_) was identified as a 4 methanol-units cluster. Its major fragments were at *m*/*z* = 579.29 for C_23_H_49_NSO_13_ and 302.14 for C_14_H_24_NSO_4_. A smaller *m*/*z* at 467.18 for C_19_H_33_NSO_10_ (compound **19** without methanol units clustered) was also observed. The nonsulfated molecule of compound **15**, pinalic-3-acid,^3,68,81−85^ has been widely observed in the environment as well as in laboratory experiments. Similarly, compounds with similar molar weight/formula to compounds **16**, **17**, **18**, and **19** have been previously observed in both laboratory experiments and the field.^11,19,22,40,42,68,69,71,72,86−89^

**Table 2:**
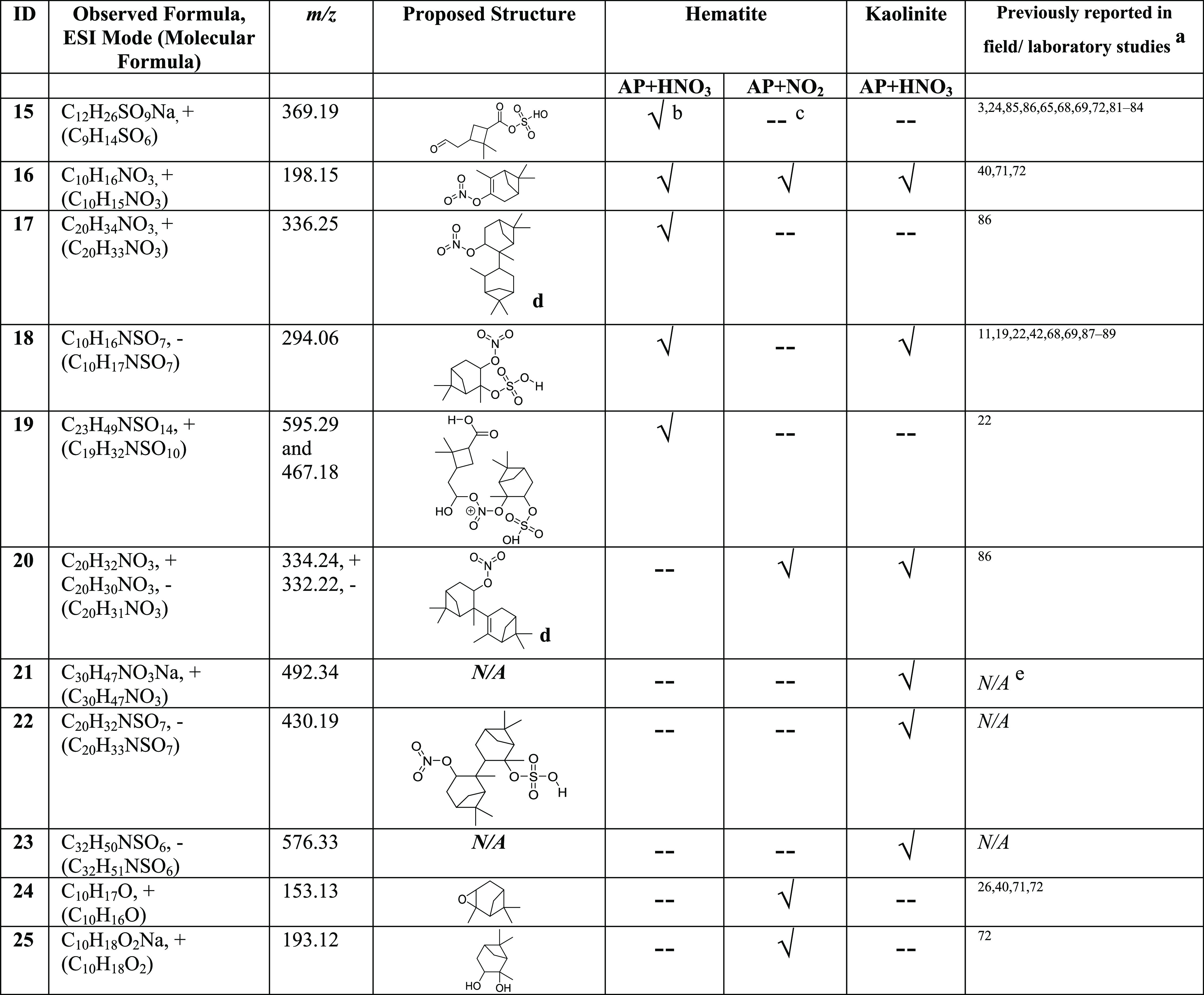
Identified Surface Products Using HRMS and MS/MS in Either Positive [M + H]+ or Negative ESI [M – H]^−^ Modes, from the Reactions of Adsorbed α-Pinene-OS with Either HNO_3_ or NO_2_ on Mineral Surfaces^a^^b^^c^^d^^e^

a One or more of the compound, molecular formula, and *m*/*z* has been reported.

b √ = Detected in HRMS.

c -- = Not detected in HRMS.

d Best matched structure with HRMS, MS/MS data, and molecular formula.

e *N/A* = Not available.

Despite wide observations of pinalic-3-acid in the environment, the mechanistic details of how this compound forms, as well as that of compound **15**, are unclear. Pinalic-3-acid forms via the ozonolysis of α-pinene. In the current study, it can be speculated that HNO_3_/adsorbed nitrates on hematite may induce the formation from the OS present on the hematite surface. Given that HSO_4_^–^ is a good leaving group,^90^ the formation of sulfated pinalic-3-acid (**15**) can occur either via oxidation of sulfated α-pinene, or sulfation of formed pinalic-3-acid by the surface-adsorbed sulfate species. Further research studies are recommended to better understand the underlying mechanisms of formation of compound **15** on mineral surfaces in the presence of HNO_3_.

**Scheme 2:**
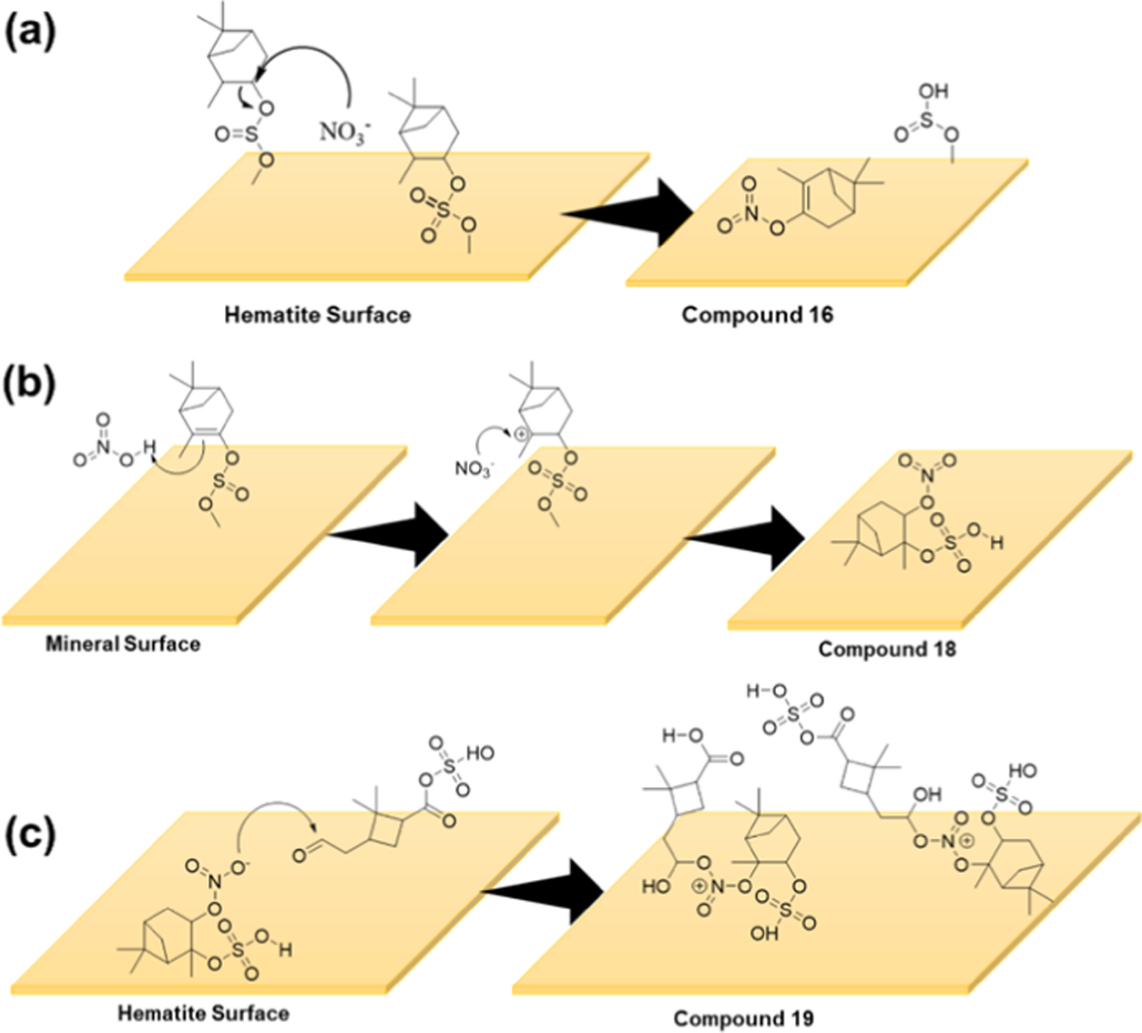
Proposed Formation Pathways for Compounds Identified in HRMS: (a) Compound **15**, (b) Compound **16**, (c) Compound **18**, and (d) Compound **19** on Mineral Surfaces

A smaller quantity of compound **16** was observed in the study. Compound **16** was previously observed in our studies forming from reactions of α-pinene with adsorbed nitrates on hematite surfaces.^40^ However, here, nitrate is likely to act as a nucleophile on either compound **1** or **2** followed by the leaving of sulfate/sulfite group as their respective acid, resulting in a C=C on compound **16** (Scheme 2). Sulfate species are known to be good leaving groups.^90^

Formation of compound **17** suggests that α-pinene-OS may undergo dimerization as well. Both α- and β-pinene dimerization has previously been studied.^91,92^ We speculate this is due to the interaction of surface hydroxyl groups with pi hydrogens in the α-pinene moiety of compounds **4**, **6**, and **16**, a carbocation forms and undergoes dimerization with compound **4** or **6** on the surface followed by elimination of H_2_SO_3_/H_2_SO_4_. However, the exact formation pathway remains unclear. Moreover, it is worth investigating whether their formation is possible at low α-pinene concentrations.

The formation of compound **18** is proposed to occur via the addition of an HNO_3_ molecule on the double bond of either compound **4** or **6**. It is expected that the sulfite group in compound **4** is oxidized by HNO_3_ molecules in the system. Compound **19** likely forms via reactions between compound **15** (or pinalic-3-acid) and compound **18**. The nitrate group in compound **18** may react with either −COOSO_3_ (−COOH in pinalic-3-acid) or aldehyde end. However, we propose the reaction undergoes via aldehyde end due to the formation of fragment 302.14 for C_14_H_24_NSO_4_. This fragment may be possible if the reaction occurs via the aldehyde end. A smaller peak at *m*/*z* = 547.14 for C_19_H_33_NS_2_O_13_ was also observed, suggesting the presence of two sulfate groups in the same molecule. However, it was not listed as a separate compound due to the lower occurrence. Despite the fact that our experimental evidence suggests the possible formation pathway for Compound **19** is as shown in Scheme 2c, given the nitrate esters are usually considered to be weak nucleophiles, a more detailed investigation of high molecular weight NOS formation, such as Compound **19**, is warranted.

**Figure 5:**
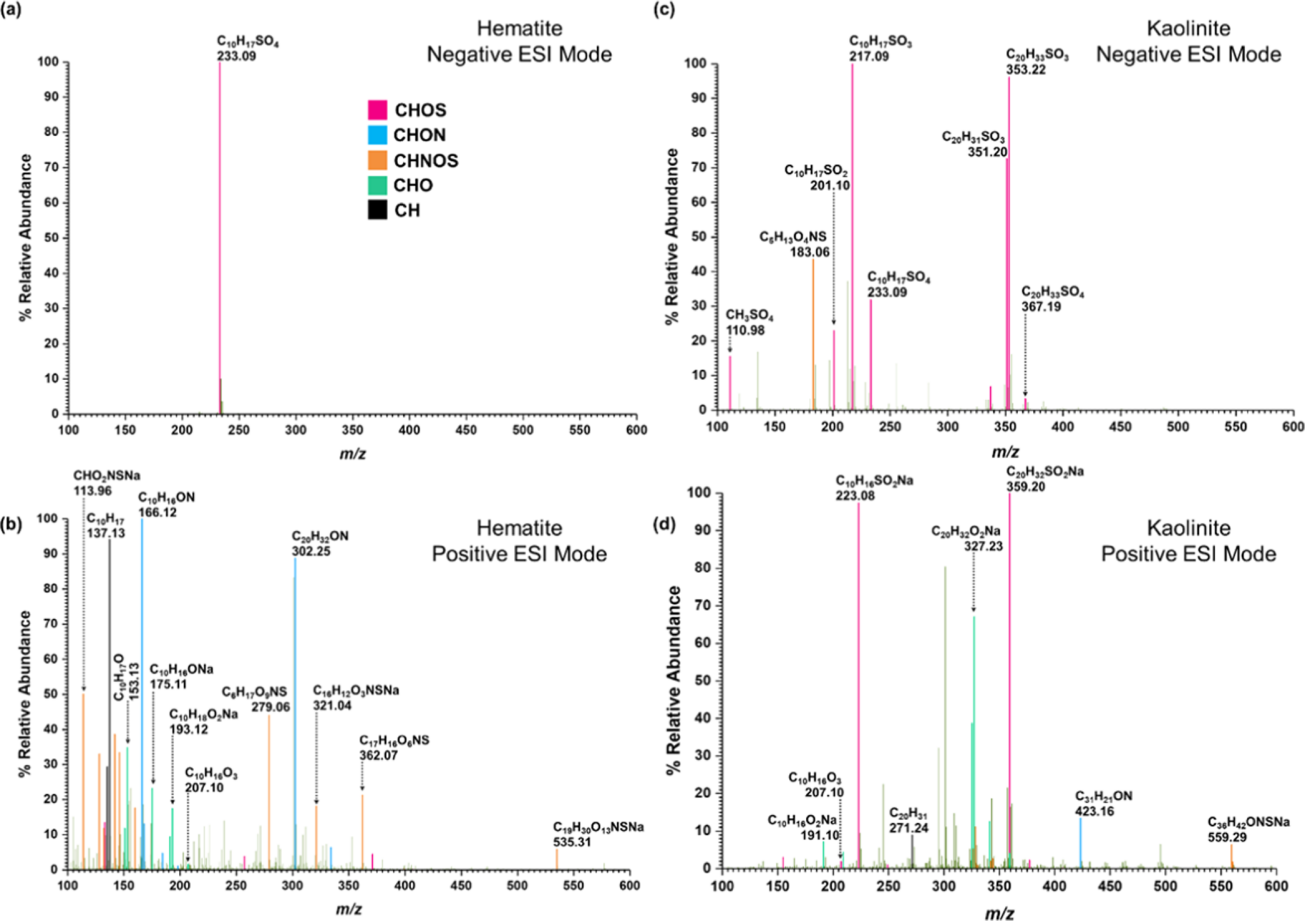
HRMS patterns in both positive [M + H]^+^ and negative [M – H]^−^ ESI modes of surface-bound products from the reactions of adsorbed α-pinene-OS with NO_2_(gas) on (a, b) hematite and (c, d) kaolinite. Confirmed peaks are color-coded according to heteroatoms. Pink box solid—CHOS; blue box solid—CHON; orange box solid—CHNOS; green box solid—CHO; black box solid—CH.

Five α-pinene-OS products were observed on kaolinite surfaces after exposure to HNO_3_ along with three ON and two NOSs. The OS are compounds **1**, **2**, **3**, **8**, and **9**. The ON, compound **20** (C_20_H_32_NO_3_, *m*/*z* = 334.24, pos and C_20_H_30_NO_3_, *m*/*z* = 332.22, neg), was identified. Another possible ON, compound **21** (C_30_H_47_NO_3_Na, *m*/*z* = 492.34), with three α-pinene moieties was identified in positive-ESI mode. Compound **21** produced several key fragments. Among these are 460.35 for C_30_H_47_ONNa, 438.37 for C_30_H_48_ON, and 324.23 for C_20_H_31_ONNa. Compound **18** (C_10_H_16_NSO_7_, *m*/*z* = 294.06) was identified in the neg-ESI mode with a major fragment at 278.07 for C_10_H_16_NSO_6_. Another NOS, compound **22** (C_20_H_32_NSO_7_, *m*/*z* = 430.19), in the neg-ESI mode produced a series of fragments at 428.18 (C_20_H_30_NSO_7_), 412.18 (C_20_H_30_NSO_6_), and 396.19 (C_20_H_30_NSO_5_). A major peak was observed at 576.33 with the formula of C_32_H_50_NSO_6_, and its fragments at 456.20 for C_29_H_30_NSO_2_, 440.21 for C_29_H_30_NSO, and 351.20 for C_20_H_31_SO_3_ were assigned to belong to the same compound (**23**). The formula or the structure of compound **23** was not identified in these studies. Many studies have reported the formation of high molecular weight ON, OS, and NOS in the environment.^11,19,22,68,69,86−88^ Similar to these studies, most interesting is that in this current study, the formation of a unique mixture of SOAs on these surfaces underscores the complexity of these reactions. Furthermore, highlighting the importance of mineral surfaces, formation of a C_9_ compound (**15**) facilitating further SOA formation of a C_19_ compound (**19**) is observed only with hematite surfaces in the presence of HNO_3_. Hematite and kaolinite surfaces represent both redox-active minerals and common clay minerals, respectively. The differences in the surface chemistry reflect the different surface properties—surface structure, surface hydroxyl group density, redox active sites, and specific surface planes. The details of these different properties and how they play a role in the reaction mechanism have yet to be fully discerned (Figure 5).

### Product Formation from the Reactions of Adsorbed α-Pinene-OS with Gas-Phase NO_2_

Reactions of NO_2_ with adsorbed α-pinene-OS on hematite surfaces produced a series of compounds. Some of these compounds were also observed during reactions of α-pinene-OS with HNO_3_ and others are unique. Some of these differences may arise from the lower acidity resulting in the mineral surface upon exposure to NO_2_ than when exposed to HNO_3_ as acid-catalyzed reactions can induce formation of products in the presence of HNO_3_ vapor. Among these were two OS, compounds **2** and **3**, two ON, compounds **16** and **20**, and four oxidized α-pinene derivatives, compounds **11**, **13**, **24** (C_10_H_17_O, *m*/*z* = 153.13 and C_10_H_16_ONa, *m*/*z* = 175.11, α-pinene oxide), and **25** (C_10_H_18_O_2_Na, *m*/*z* = 193.12, α-pinene diol). Compounds **2** and **3** were identified in both negative and positive ESIs modes. Furthermore, a range of peaks containing NSO groups were identified in positive-ESI mode, suggesting the formation of NOS during these reactions. These peaks were 535.31 for C_19_H_30_NSO_13_Na, 362.07 for C_17_H_16_NSO_6_, 321.04 for C_16_H_12_NSO_3_Na, 279.06 for C_6_H_17_NSO_9_, and 113.96 for CHNSO_2_Na. Although none of these peaks were assigned to a particular compound, it is important to note that both C_17_ and C_16_ compounds have been previously observed from α-pinene SOAs.^85,86,93^ Similarly, reactions of adsorbed α-pinene-OS on kaolinite surfaces with NO_2_ produced a mixture of different SOAs. The OS **1**, **2**, **3**, **7**, **9**, and **10** were observed. The oxygenated α-pinene derivatives, compounds **11**, **12**, and **13**, were also observed. However, no new products compared to α-pinene alone with sulfated kaolinite was observed. Several peaks containing NSO were observed in both neg- and pos-ESI modes. However, similar to the case of hematite, here also no peak was assigned to a particular NOS compound due to the complexity of HRMS and MS/MS data. Overall, the current study suggests the formation of a unique mixture of ON and NOS and other oxidation products from the reactions of gas-phase NO_2_ with adsorbed α-pinene-OS on mineral surfaces. Future research studies are recommended for understanding the reactions of NO_2_ with VOC-derived OS adsorbed on mineral surfaces to better understand these reaction pathways. In particular, these studies can benefit from computational analyses. Quantification of identified surface products was not conducted in the current study due to the lack of availability of suitable standards.

## Conclusions and Atmospheric Implications

The reactions of α-pinene on sulfated hematite and kaolinite surfaces at 296 K yield several different OS and oxidation products of α-pinene. Kaolinite surfaces produce pinonaldehyde and several OS compounds with a pinonaldehyde moiety, whereas all OS identified on hematite retained the α-pinene moiety, highlighting the underlying differences in the role of mineralogy and surface-specific chemistry. The adsorbed α-pinene-OS reacts with HNO_3_ or NO_2_ to produce more OS, multiple ON, and NOS products on surfaces. The proposed formation pathways of these OS occur via an adsorbed sulfite/sulfate species. The formation of ON and NOS compounds in the presence of HNO_3_ suggests the addition of HNO_3_, nucleophilic attacks by nitrates on OS, and reactions between ON and OS on the surface. Among other products, C_10_H_17_NSO_7_, commonly known as NOS-295, was observed on both surfaces from reactions of OS with HNO_3_. On hematite, OS (C_9_H_14_SO_6_) and NOS (C_19_H_32_NSO_10_) were observed, indicating the formation of C_9_ compounds in the presence of HNO_3_, thus underscoring the specific roles of mineral surfaces. The majority of surface products identified from the reactions of OS with NO_2_ on both surfaces were either OS or oxidized α-pinene derivatives, along with evidence of the formation of NOS.

Overall, this study shows how mineral dust aerosol surface can interact with inorganic and organic gas-phase compounds to yield a wide range of compounds important for secondary organic aerosol surfaces. These data show that surfaces can interact with gas-phase SO_2_ that then lead to OS surface products in the presence of ubiquitous organic compounds such as α-pinene and then further can yield ON and NOS compounds when gas-phase nitrogen oxides are present. Most notable is that the specific products that form are mineral specific. Additionally, some of the SOAs observed in this study have been repeatedly observed in field studies; yet, formation pathways are poorly understood. In the environment, VOCs such as α-pinene are ubiquitous. During dust transport and urban dust episodes, these VOCs and trace gas pollutants such as NO_2_ and SO_2_ can interact with each other, forming more highly functional, less volatile compounds as shown here on two different mineral surfaces under dry conditions. As water plays an important role in heterogeneous chemistry,^53^ it is important to further investigate how adsorbed water molecules impact these reactions. This study suggests heterogeneous reactions are possible pathways for the formation of these OS, ON, and NOS compounds but we also note the importance of extending this to different environmental conditions (e.g., relative humidity and solar radiation) on these formation pathways.

## Supporting Information Available

FTIR spectra of sulfated mineral surfaces (Figure S1); vibrational frequencies of different adsorbed sulfur-containing species on hematite and kaolinite (Table S1); and MS/MS analysis of parent peaks for the identified compounds (Table S2) (PDF)
