# Supplementary material for: Heterogeneous Formation of Organonitrates (ON) and Nitroxy-Organosulfates (NOS) from Adsorbed α-Pinene-Derived Organosulfates (OS) on Mineral Surfaces
Source: ACS Earth Space Chem. 2022 Nov 29;6(12):3017–30. doi: 10.1021/acsearthspacechem.2c00259 (PMC9762235; doi:10.1021/acsearthspacechem.2c00259)
Supplement: Supplementary file 1 — sp2c00259_si_001.pdf [file sp2c00259_si_001.pdf]

Supporting Information

for

Heterogeneous Formation of Organonitrates (ON) and Nitroxy-Organosulfates (NOS) from Adsorbed Alpha-Pinene Derived Organosulfates (OS) on Mineral Surfaces

Eshani Hettiarachchi and Vicki H. Grassian*

Department of Chemistry and Biochemistry, University of California San Diego, 9500 Gilman Drive, La Jolla, CA, 92093

*Author to whom correspondence should be addressed (vhgrassian@ucsd.edu)

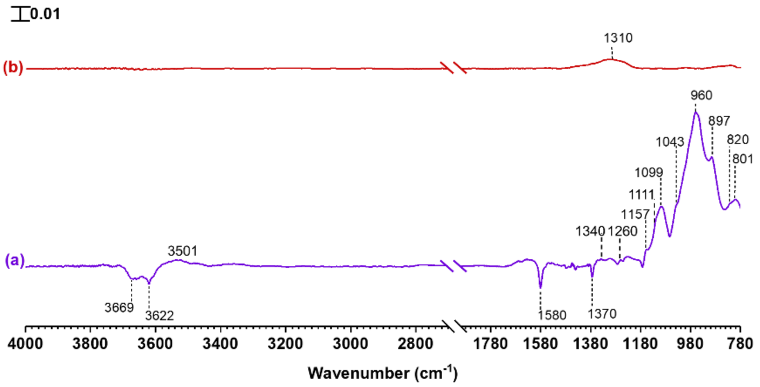

Figure S 1: FTIR spectra of sulfated (a) hematite, and (b) kaolinite surfaces. The intensity scale is shown in the top left corner.

Table S 1: Vibrational frequencies of different adsorbed sulfur-containing species on hematite and kaolinite. [1–5]

**Molecular Species**		**Hematite**	**Kaolinite**
**SO_2**		1340, 1353, 1360	
**HSO_3^-**		1043, 1099	
**SO_3^{2-}**			
	Monodentate	960, 1043	
	Bidentate	820, 897	
**SO_4^{2-} (Several coordination modes)**		1157, 1111, 1260	1310
**SO_2•H_2O complex O-H region**		3501	
**Loss of surface O-H groups**		3622, 3659, 3669	

Table S 2: MS/MS Analysis of Parent Peaks for Identified Compounds

ID	Parent Peak, Observed formula, ESI mode	Fragments	Formula
**01**	217.09, $C_{10}H_{17}SO_3$, Neg	201.10	$C_{10}H_{17}SO_2$
		79.95	SO_3
**02 & 03**	233.09, $C_{10}H_{17}SO_4$, Neg	79.95	SO_3
		80.97	HSO_3
		96.96	HSO_4
**04**	215.07, $C_{10}H_{15}SO_3$, Neg	80.97	HSO_3
**05**	249.08, $C_{10}H_{17}SO_5$, Neg	137.13	$C_{10}H_{17}$
**06**	231.07, $C_{10}H_{15}SO_4$, Neg	135.12	$C_{10}H_{15}$
**07**	415.18, $C_{20}H_{31}SO_7$, Neg	351.20	$C_{20}H_{31}SO_3$
		399.19	$C_{20}H_{31}SO_6$
		383.19	$C_{20}H_{31}SO_5$
		367.20	$C_{20}H_{31}SO_4$
		333.19	$C_{20}H_{29}SO_2$
**08**	551.31, $C_{30}H_{47}SO_7$, Neg	503.32	$C_{30}H_{47}SO_4$
		487.33	$C_{30}H_{47}SO_3$
**09**	249.09, $C_{10}H_{17}SO_5$, Pos	223.08	$C_{10}H_{16}SO_2Na$
		191.10	$C_{10}H_{16}O_2$
**10**	407.18, $C_{20}H_{32}SO_5Na$, Pos	391.19	$C_{20}H_{32}SO_4Na$
		375.20	$C_{20}H_{32}SO_3Na$
		359.20	$C_{20}H_{32}SO_2Na$
**11**	191.10, $C_{10}H_{16}O_2Na$, Pos		
**12**	359.22, $C_{20}H_{32}O_4Na$, Pos	343.22	$C_{20}H_{32}O_3Na$
		327.23	$C_{20}H_{32}O_2Na$
**13**	207.10, $C_{10}H_{16}O_3Na$, Pos	--	--
**14**	375.21, $C_{20}H_{32}O_5Na$, Pos	--	--
**15**	369.12, $C_{12}H_{26}SO_9Na$, Pos	367.10	$C_{12}H_{24}SO_9Na$
		365.09	$C_{12}H_{22}SO_9Na$
		228.01	$C_9H_8SO_5$
**16**	198.15, $C_{10}H_{15}NO_3$, Pos	166.12	$C_{10}H_{16}NO$
**17**	336.25, $C_{20}H_{34}NO_3$, Pos	320.26	$C_{20}H_{34}NO_2$
	Frag. 320.26	302.25	$C_{20}H_{32}ON$
		168.14	$C_{10}H_{18}ON$
		135.12	$C_{10}H_{15}$
		109.10	C_8H_{13}
		276.23	$C_{18}H_{30}ON$
		248.20	$C_{16}H_{26}ON$
		194.15	$C_{12}H_{20}ON$
**18**	294.06, $C_{10}H_{16}NSO_7$, Neg	278.07	$C_{10}H_{16}NSO_6$
**19**	595.29, $C_{23}H_{49}NSO_{14}$, Pos	579.29	$C_{23}H_{49}NSO_{13}$
	Frag. 579.29	547.26	$C_{22}H_{45}NSO_{12}$
		339.08	$C_{17}H_{16}O_6Na$
		467.10	$C_{22}H_{22}NSO_7Na$
		302.14	$C_{14}H_{24}NSO_4$

**20**	334.24, $C_{20}H_{32}NO_3$, Pos	318.24	$C_{20}H_{32}NO_2$
		302.25	$C_{20}H_{32}ON$
	332.22, $C_{20}H_{30}NO_3$, Neg	316.23	$C_{20}H_{30}NO_2$
		314.21	$C_{20}H_{28}NO_2$
**21**	492.34, $C_{30}H_{47}NO_3Na$, Pos	460.35	$C_{30}H_{47}ONNa$
		438.37	$C_{30}H_{48}ON$
		324.23	$C_{20}H_{31}ONNa$
**22**	430.19, $C_{20}H_{32}NSO_7$, Neg	428.18	$C_{20}H_{30}NSO_7$
		412.18	$C_{20}H_{30}NSO_6$
		396.19	$C_{20}H_{30}NSO_5$
**23**	576.33, $C_{32}H_{50}NSO_6$, Neg	456.20	$C_{29}H_{30}NSO_2$
		440.21	$C_{29}H_{30}NSO$
		351.20	$C_{20}H_{31}SO_3$
**24**	153.13, $C_{10}H_{17}O$, Pos	137.13	$C_{10}H_{17}$
	175.11, $C_{10}H_{16}ONa$, Pos	137.13	$C_{10}H_{17}$
**25**	193.12, $C_{10}H_{18}O_2Na$, Pos	137.13	$C_{10}H_{15}$

References

(1) Nanayakkara, C. E.; Pettibone, J.; Grassian, V. H. Sulfur Dioxide Adsorption and Photooxidation on Isotopically-Labeled Titanium Dioxide Nanoparticle Surfaces: Roles of Surface Hydroxyl Groups and Adsorbed Water in the Formation and Stability of Adsorbed Sulfite and Sulfate. *Phys. Chem. Chem. Phys.* **2012**, *14* (19), 6957–6966. https://doi.org/10.1039/c2cp23684b.

(2) Wang, T.; Liu, Y.; Deng, Y.; Fu, H.; Zhang, L.; Chen, J. Adsorption of SO_2 on Mineral Dust Particles Influenced by Atmospheric Moisture. *Atmos. Environ.* **2018**, *191*, 153–161. https://doi.org/10.1016/j.atmosenv.2018.08.008.

(3) Goodman, A. L.; Li, P.; Usher, C. R.; Grassian, V. H. Heterogeneous Uptake of Sulfur Dioxide On Aluminum and Magnesium Oxide Particles. *J. Phys. Chem. A* **2001**, *105* (25), 6109–6120. https://doi.org/10.1021/jp004423z.

(4) Huang, L.; Frank, E. S.; Riahi, S.; Tobias, D. J.; Grassian, V. H. Adsorption of Constitutional Isomers of Cyclic Monoterpenes on Hydroxylated Silica Surfaces. *J. Chem. Phys.* **2021**, *154* (12), 124703. https://doi.org/10.1063/5.0042467.

(5) Ho, J.; Psciuk, B. T.; Chase, H. M.; Rudshteyn, B.; Upshur, M. A.; Fu, L.; Thomson, R. J.; Wang, H. F.; Geiger, F. M.; Batista, V. S. Sum Frequency Generation Spectroscopy and Molecular Dynamics Simulations Reveal a Rotationally Fluid Adsorption State of α-Pinene on Silica. *J. Phys. Chem. C* **2016**, *120* (23), 12578–12589. https://doi.org/10.1021/acs.jpcc.6b03158.
